# Disulfiram Suppressed Peritendinous Fibrosis Through Inhibiting Macrophage Accumulation and Its Pro-inflammatory Properties in Tendon Bone Healing

**DOI:** 10.3389/fbioe.2022.823933

**Published:** 2022-03-08

**Authors:** Qi Zhou, Wei Wang, Fujun Yang, Hao Wang, Xiaodong Zhao, Yiqin Zhou, Peiliang Fu, Yaozeng Xu

**Affiliations:** ^1^ Department of Orthopedics, the First Affiliated Hospital of Soochow University, Suzhou, China; ^2^ Department of Orthopedics, Shanghai Changzheng Hospital, Naval Medical University, Shanghai, China; ^3^ The Fifth People’s Hospital of Zunyi, Zunyi, China; ^4^ Department of Orthopaedics, Shanghai Public Health Clinical Center, Fudan University, Shanghai, China; ^5^ Department of Orthopaedics, Weifang Traditional Chinese Hospital, Weifang, China

**Keywords:** macrophage, tendon–bone injury, disulfiram, gasdermin D, fibrosis

## Abstract

The communication between macrophages and tendon cells plays a critical role in regulating the tendon-healing process. However, the potential mechanisms through which macrophages can control peritendinous fibrosis are unknown. Our data showed a strong pro-inflammatory phenotype of macrophages after a mouse tendon–bone injury. Moreover, by using a small-molecule compound library, we identified an aldehyde dehydrogenase inhibitor, disulfiram (DSF), which can significantly promote the transition of macrophage from M1 to M2 phenotype and decrease macrophage pro-inflammatory phenotype. Mechanistically, DSF targets gasdermin D (GSDMD) to attenuate macrophage cell pyroptosis, interleukin-1β, and high mobility group box 1 protein release. These pro-inflammatory cytokines and damage-associated molecular patterns are essential for regulating tenocyte and fibroblast proliferation, migration, and fibrotic activity. Deficiency or inhibition of GSDMD significantly suppressed peritendinous fibrosis formation around the injured tendon and was accompanied by increased regenerated bone and fibrocartilage compared with the wild-type littermates. Collectively, these findings reveal a novel pathway of GSDMD-dependent macrophage cell pyroptosis in remodeling fibrogenesis in tendon–bone injury. Thus, GSDMD may represent a potential therapeutic target in tendon–bone healing.

## Introduction

Peritendinous fibrosis is a critical complication arising after tendon injury (TI), which is primarily identified as excessive extracellular matrix (ECM) deposition and fibroblast cell activation (Manske, 2005; Juneja et al., 2013). Presently, the imbalance between ECM synthesis and degradation has been established as the leading cause of impaired tendon function and increased risk of repair after clinical surgery (Zheng et al., 2019). The transdifferentiation and proliferation of fibroblasts are the major source of ECM and can be regulated by a series of differential cytokine release in adhesive tendon tissues, including that of transforming growth factor (TGF)-β, fibroblast growth factor-2, connective tissue growth factor, and vascular endothelial growth factor (Darby et al., 2016). In addition, while the resident tissue fibroblasts have been identified as sensitive to intrinsic mechanical and biophysical cues in tendon–bone injury (Zheng et al., 2017; Yao et al., 2019), the initiation of immune response in this process remains poorly understood.

The earliest cellular response following the surgical implantation of a tendon graft in a bone tunnel is accumulation of the various inflammatory cells (Kawamura et al., 2005). In general, the main effector myeloid cells, including monocytes and macrophages, are the first cells that are activated in response to the TI. M1 macrophages can produce different pro-inflammatory cytokines such as interleukin-1β (IL-1β) and tumor necrosis factor-α (TNF-α) during immune activation. These pro-inflammatory cytokines not only have an increased level in the early process of TI but also modulate tendon–bone response after magnetic stimulation. A number of previous studies have indicated that macrophages can negatively affect the process as macrophage depletion can significantly improve both the morphological and biomechanical properties at the healing tendon–bone interface (Cui et al., 2019). Moreover, during the successful tissue–biomaterial integration, M1 macrophages can effectively polarize into M2 macrophages (Klopfleisch, 2016), which express different genes and attain distinct functional states. M2 macrophages are anti-inflammatory, are antiparasitic, and can promote tissue repair. However, the precise mechanisms that can regulate M2 macrophage polarization and function in the tendon–bone injury are unknown.

Drug repurposing has been recently identified as a cost-effective approach to bring approved drugs rapidly in clinical treatment (Alvarez et al., 2021). In prior investigation on repurposed drugs, such as aspirin, they have been demonstrated to promote tenogenic differentiation of the tendon stem cells and to facilitate tendinopathy healing (Wang et al., 2020). Disulfiram (DSF) is an old alcohol-abuse drug that was approved by the US Food and Drug Administration (FDA) in 1949 (Bista et al., 2017). Except for its role as an acetaldehyde dehydrogenase, recent studies have revealed its role in various diseases, including antitumor activity and resistance to sepsis and obesity (Skrott et al., 2017; Bernier et al., 2020; Klimiankou and Skokowa, 2021). However, the role of DSF on macrophage is unknown. In TI, the functional phenotype change of M1 and M2 exhibits a phagocytic and pro-inflammatory function, which plays an important role in the healing process (Chamberlain et al., 2021; Yu et al., 2021). Furthermore, the reduced macrophage-mediated inflammation in the injured tendon through M1-to-M2 transmission significantly inhibits adhesion formation during tendon healing (Dong et al., 2021). Consequently, it is desirable to explore the effect of DSF on the immunoregulatory potential of macrophage in tendon–bone injury.

In the present study, we have identified a small molecular compound, DSF, which can significantly promote the transition of macrophage from the M1 to M2 phenotype. We further uncover a novel mechanism through which DSF can target gasdermin D (GSDMD) to inhibit macrophage cell pyroptosis in the tendon bone injury and a newly identified GSDMD-dependent cell interaction between immune cells and resident tendon cells. We have also explored the therapeutic potential of targeting this pathway in the context of TI.

## Materials and Methods

### Reagents and Antibodies

The cell culture medium including Dulbecco’s Modified Eagle’s Medium (DMEM; Cat#: 10-013-CVR, Corning, New York, NY, United States) and supplements including penicillin and streptomycin (Cat#: 15140-122, Gibco, Waltham, MA, United States) were purchased from Life Science Technologies (Rockville, MD, United States). Antibodies to GSDMD (ab219800), fibronectin (FN) (ab2413), collagen I (ab34710), α-smooth muscle actin (α-SMA) (55135-1-AP), IL-1β (ab9722), high mobility group box 1 (HMGB1) (ab18256) were obtained from Abcam (Cambridge, MA, United States). Anti-caspase-11 (17D9, C1354) was from Sigma-Aldrich (Burlington, MA, United States). Anti-caspase-1 (AC-20B-00420-C100) was from AdipoGen (San Diego, CA, United States). β-Actin antibody (3700S) was purchased from Cell Signaling Technology (Danvers, MA, United States). Anti-IL-1α (sc-12741) was purchased from Santa Cruz (TX, United States). Fetal bovine serum (FBS) (Cat#: 26140-079, Gibco, NY, United States) was purchased from Life Science Technologies and heat-inactivated before utilization. M-CSF was obtained from Miltenyi Biotec (130-094-129, Westphalia, Germany). Recombinant mouse INF-γ (C600059) and IL-4 (C600050) were purchased from Sangon Biotech (Shanghai, China). Fifty-four drug libraries were directly obtained from Topscience (L2130, Shanghai, China). LPS (L2630) and DSF (PHR-1690) were purchased from Sigma-Aldrich (Sigma, Merck, Darmstadt, Germany).

### Animals

All animal experiments in this study were approved by the Navy Medical University Animal Care and Use Committee. Pain and discomfort are minimized based on standard animal care guidelines. Male mice (6 weeks, SLRC Laboratory, Shanghai, China) were anesthetized by an initial intraperitoneal injection of Avertin (100 mg kg^−1^). The Achilles tendon of the left leg was then exposed and released from the calcaneum. Thereafter, the tendon was cut at its insertion on the bone. The enthesis was destroyed by a burr. An 18G sterile needle was used to make two tunnels through the calcaneum, and a nonabsorbable suture was used to attach the tendon back to the bone. Then the wound was closed, and the mice were allowed to recover. Mice were sacrificed 30 days after surgery. For flexor tendon surgery, mice underwent complete transection and repair of the flexor digitorum longus (FDL) tendon in the right hind paw as previously reported (Loiselle et al., 2009).

### Histological Evaluation

The excess soft tissues were isolated from the mice and kept the repair site complete. The specimens were fixed in 4% paraformaldehyde (PFA) for 24 h and then decalcified in Plank-Rychlo decalcifying fluid at room temperature for 16 days. The specimens were embedded in paraffin, and thereafter, the slices were cut and stained with hematoxylin and eosin (H&E). The histological evaluation was performed according to the histological scoring system (Jiang et al., 2014).

### Exosome Purification

Exosome purification was performed as previously described (Cui et al., 2019). Briefly, the bone-marrow-derived macrophages (BMDMs) were cultured for 72 h. The debris and dead cells in the cultured medium were removed by centrifugation at 1,000 × *g* for 10 min at 4°C. After filtration through a 2-μm filter, the medium was subjected to ultracentrifugation at 100,000 × *g* for 6 h at 4°C. The pellets were washed with PBS and then centrifuged at 100,000 × *g* for 20 min at 4°C. An exosome-containing pellet was collected and resuspended in PBS. The particle size and concentration of exosome were determined using network address translation (NTA) with ZetaView PMX 120 (Particle Metrix).

### Confocal Immunofluorescent Imaging

The tendon sections from the frozen optimum cutting temperature compound (OCT)-embedded tissues were fixed with 1:1 acetone/methanol and stained with H&E. The cells were fixed in 2% PFA for 15 min at 4°C. Thereafter, the cells were washed with PBS only once and permeabilized in 0.1% Triton X-100 at 4°C for 10 min. The cells were then blocked with 3% goat serum plus 3% FBS at 4°C for 1 h. F4/80 and α-SMA were added overnight at 4°C. The cells were washed with PBS only once; the cells were then incubated with the secondary antibody for 1 h at 4°C and gently washed three times with PBS before imaging. Imaging was performed on a custom modified Olympus FV3000 laser scanning microscope equipped with a ×60 oil immersion lens.

### Flow Cytometry Analysis

For the surface and cytokine staining, 100 μl of blood was harvested and blocked with an Fc blocker (BioLegend) for 30 min at 4°C. The cells were then resuspended in a 50 μl fluorescence activating cell sorter (FACS) buffer (1× PBS with 0.5% FBS, 2 mM ethylenediaminetetraacetic acid) with diluted CD86 and CD206. iNOS and TNF-α antibody were added by Foxp3/Transcription Factor Staining Buffer Set (eBioscience) according to manufacturer’s protocol. For cell cycle analysis, the cells were collected and suspended in pre-cold absolute ethanol for overnight at 4°C. Thereafter, the cells were stained with propidium iodide (PI) for 30 min at dark condition. The data were acquired on a BD LSRFortessa X-20 cell analyzer (BD Biosciences) and analyzed using the FlowJo software (Tree Star, Inc.).

### Cell Cultures

NIH/3T3 cell lines were purchased from the Cell Bank of Type Culture Collection, Chinese Academy of Sciences (CAS, Shanghai, China), and were cultured in DMEM supplemented with 10% FBS. For BMDMs, the mice were sacrificed by cervical vertebra dissection, followed by soaking in 75% alcohol for 3 min. The femur and tibia were separated on a sterile bench. The lumen was rinsed with PBS solution containing 3% FBS, and bone fragments and meat scraps were removed with a 70-µm filter. The cells were then centrifuged at 400 × *g* of 4°C for 10 min. Thereafter, the red blood cells were subjected to lysis by red blood cell lysis buffer. The cells were suspended in 1640 medium with 10% FBS. A total of 1 × 10^7^ cells were cultured in a T25 flask overnight, and any contaminating cells such as monocytes and fibroblasts were removed. After centrifugation at 400 × *g* for 5 min, cells were resuspended in 1640 medium (containing 10% FBS and penicillin streptomycin). The cells were then treated with the macrophage-stimulating factor (M-CSF) at 10 ng/ml and transferred in a new T25 culture flask. The incubation was continued, and the medium was changed every 3 days, but the M-CSF concentration was kept constant. On the seventh day, the cells were collected and tested for purity.

### Western Blotting

The tendons or cells were lysed in cell lysis buffer (Cell Signaling Technology, #9803) and supplemented with proteinase inhibitor at a dilution of 1:25. The protein concentrations were determined by using a BCA protein assay kit. For western blot analysis, equal amounts of protein were heat-denatured in the presence of a reducing agent and separated on 10% SDS-PAGE and transferred to PVDF membranes (Millipore, IPVH00010). The various antibodies used for western blot have been mentioned in the figure legend. The bands were detected using ECL Plus (Tanon, 180-5001) using the ChemiDoc Imaging System (AI600). The data were analyzed by ImageJ and normalized with control (as mentioned in the figure legend).

### Cell Proliferation Assay

NIH/3T3 fibroblasts were seeded at a density of 2 × 10^3^ per well into 96-well plates and were then treated with BMDM exosomes or pretreated with DSF (5 μM). The cell proliferation was assessed using the BeyoClick™ 5-ethynyl-2′-deoxyuridine (EdU) cell proliferation kit with TMB (Beyotime Biotechnology, Jiangsu, China). This method is based on EdU acting as a novel alternative for 5-bromo-2′-deoxyuridine (BrdU) assay to directly measure the DNA synthesis or S-phase synthesis of a cell cycle *via* the reaction with fluorescent azide. The absorbance was measured at 630 OD. The experiment was performed in triplicate.

### Wound Healing

NIH/3T3 fibroblasts were cultured and marked a line at the bottom. After the cells were found to cover the bottom of the plate, a 1 ml pipette tip was used to make cell scratches perpendicular to the well plate, and it was ensured that the width of each scratch remained the same. The cell culture medium was aspirated, and the well plate was rinsed three times with PBS to wash away the cell debris generated by the scratch. Thereafter, a serum-free medium was added, and pictures were taken and recorded. The culture plate was placed into the incubator for culture and was taken out every 4–6 h to capture the pictures. The experimental results were analyzed according to the collected picture data.

### Morphological Analysis of Macrophages

BMDMs were isolated from the mice, seeded in the six-well plate (Life Technologies), and the cells were allowed to adhere overnight at 37°C. The cells were stimulated with LPS (100 ng/ml) for 12 h after serum starvation. For inhibitor analysis, the cells were preincubated with DSF (4 μM) or DMSO (0.01%) for 1 h. The confocal images were obtained using an FV3000 confocal system (Olympus), and the data were analyzed with ImageJ (NIH, V2.0.0).

### Statistical Analyses

The results have been expressed as mean ± SEM. Unpaired Student’s *t*-tests were used to compare the means of the two different groups. One-way analysis of variance (Tukey test) was applied to compare the means of three or more groups. The Wilcoxon (Gehan) statistical test was used to analyze the survival rate.

## Results

### An Increase in Pro-inflammatory Phenotype of Macrophages and Peritendinous Fibrosis Was Observed in Mouse TI

**FIGURE 1:**
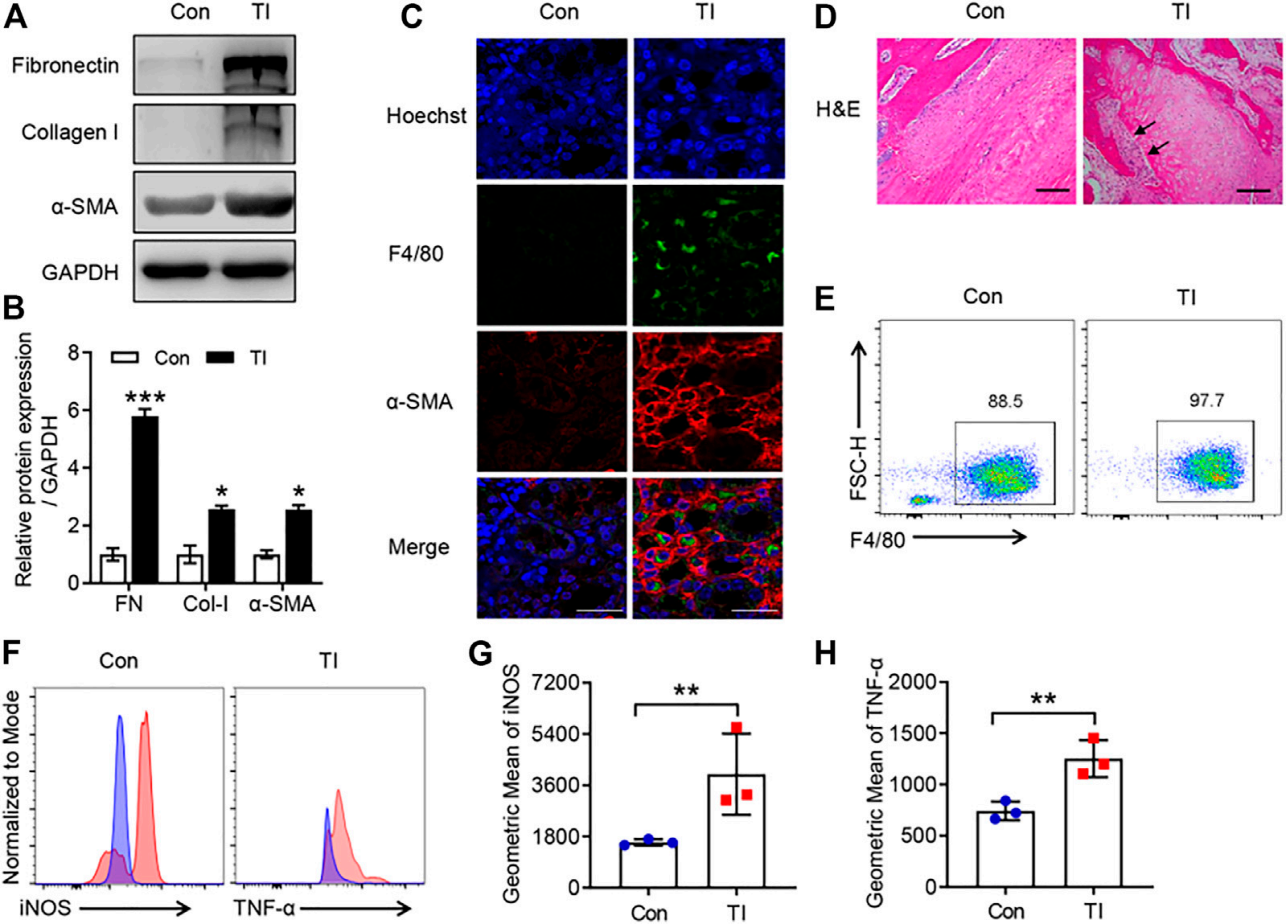
The increased peritendinous fibrosis and macrophage infiltration in TI. **(A)** Western blot analysis of FN, Col-I, and α-SMA in tendon from control (Con) and TI mice. **(B)** Quantitative analysis of FN, Col-I, and α-SMA in **A**. **(C)** Confocal analysis of F4/80 and α-SMA in the tendon section from Con and TI mice. Scale bar = 20 μm. **(D)** H&E staining in the tendon section from Con and TI mice. Scale bar = 200 μm. **(E)** Flow cytometric analysis (FACS) of M-CSF-induced macrophages (F4/80^+^) from Con and TI groups. **(F)** FACS representative analysis of iNOS and TNF-α. **(G,H)** Quantitative analysis of geometric mean of iNOS and TNF-α in E. Data are presented as the mean ± SEM (*n* = 3) **(B,G,H)**. *p < 0.05, **p < 0.01, ***p < 0.001.

Persistent immune cell activation, as well as upregulation of pro-fibrotic cytokines and ECM proteins, has been reported to be involved in fibrotic diseases (Chen et al., 2017; Pakshir and Hinz, 2018). We investigated the potential role of macrophage and ECM proteins in TI. There was a significantly increased expression of pro-fibrotic markers in the TI group compared with the control (Figure 1). The flexor tendons and surrounding tissues showed F4/80-positive staining and displayed an increased an α-SMA immunofluorescence signal in the TI group compared with the control (Figure 1C). The results of H&E also showed increased cells and cell nuclear staining in flexor tendons and surrounding tissues (Figure 1D). We then isolated BMDMs from control and TI mice. Interestingly, we found that BMDMs from the TI group showed increased expression of F4/80 compared with BMDMs from the control group (Figure 1E). The intrinsic gene expression of macrophages can effectively change and polarize into two distinct types, M1-type macrophages and M2-type macrophages (Wang et al., 2021). The intensity of iNOS and TNF-α in BMDMs was significantly increased from the TI group as compared with the control (Figure 1), thereby indicating the phenotype of M1-type macrophage in BMDM from TI mice. Collectively, these results suggested that pro-inflammatory macrophage and fibrosis were significantly activated in TI mice.

### DSF Decreased Macrophage Pro-inflammatory Phenotype and Can Promote the Transition of Macrophage From M1 to M2 Phenotype

**FIGURE 2:**
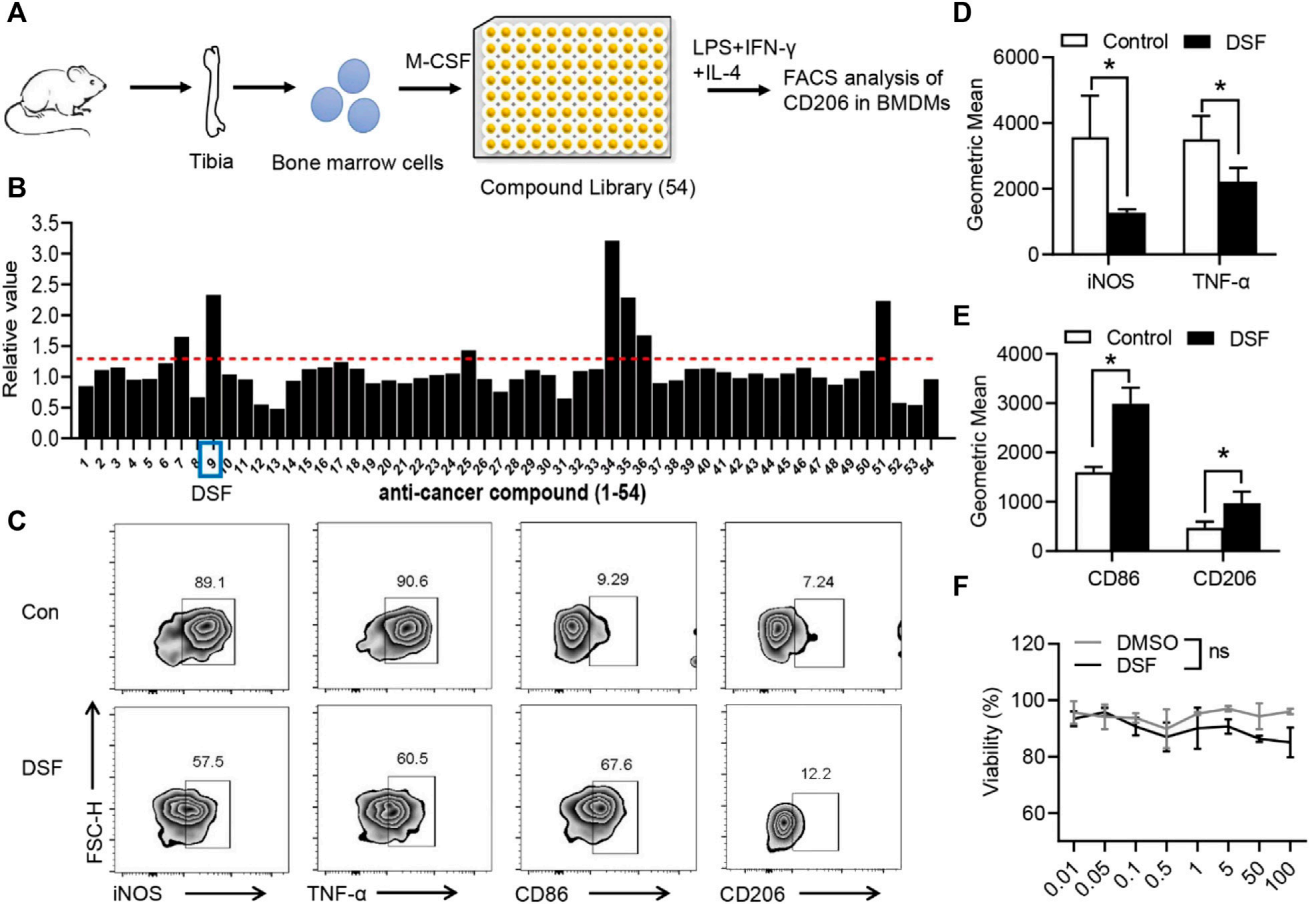
DSF significantly promotes pro-inflammatory macrophages into the M2-type phenotype. **(A)** The diagram of screening assay. Bone marrow cells were isolated from the tibia of wild-type mice. BMDMs were induced by M-CSF (50 ng/ml). Then the cells were treated with LPS (100 ng/ml), IFN-γ (20 ng/ml), and IL-4 (20 ng/ml) for 6 h and underwent FACS analysis of CD206 expression. **(B)** Quantitative analysis of the candidates in screening assay. **(C)** BMDMs were pretreated with DSF (5 μM) or DMSO (0.01%). Then the cells were treated with LPS (100 ng/ml), IFN-γ (20 ng/ml), and IL-4 (20 ng/ml) for 6 h. FACS analysis of iNOS, TNF-α, CD86, and CD206. **(D,E)** Quantitative analysis of geometric mean of iNOS **(D)** TNF-α **(D)**, CD86 **(E)** and CD206 **(E)**. **(F)** CCK-8 analysis of BMDMs after treatment with DSF at different dosages. Data are presented as the mean ± SEM (*n* = 3) **(D**,**E**,**F)**. *p < 0.05.

It has been established that the different tissue microenvironment and pathological conditions can facilitate the polarization of macrophages into the M2 type, which can then secrete the various anti-inflammatory cytokines and promote fibrocartilage regeneration, thus promoting the healing of TI (Dong et al., 2021). To identify whether the macrophage pro-inflammatory phenotype could be modulated by a pharmacological agent, we performed a screen of 54 natural small-molecular compounds to analyze their effects on CD106, a critical marker of the M2 phenotype (Figure 2A). BMDMs were isolated from the tibia of C57BL mice and pre-incubated with small-molecular compounds in a 96-well plate. BMDMs were induced by M-CSF, and cells were then treated with LPS, IFN-γ, and IL-4 for 6 h. The flow cytometry analysis showed that the ninth compound, marketed as DSF, significantly promoted the expression of CD206 (Figure 2B). We further investigated the markers of M1- and M2-type macrophages. The percentage and geometric mean of both iNOS and TNF-α were substantially suppressed by DSF (Figure 2), while the expression levels of CD86 and CD206 were increased after treatment with DSF (Figure 2). We also detected the cell viability of BMDMs by using the CCK-8 assay, and the results showed no significant differences between DMSO and DSF treatments. These findings indicated that DSF could significantly promote the M2-type macrophage transformation of BMDMs after LPS, IFN-γ, and IL-4 treatments.

### DSF Suppressed Peritendinous Fibrosis in TI

**FIGURE 3:**
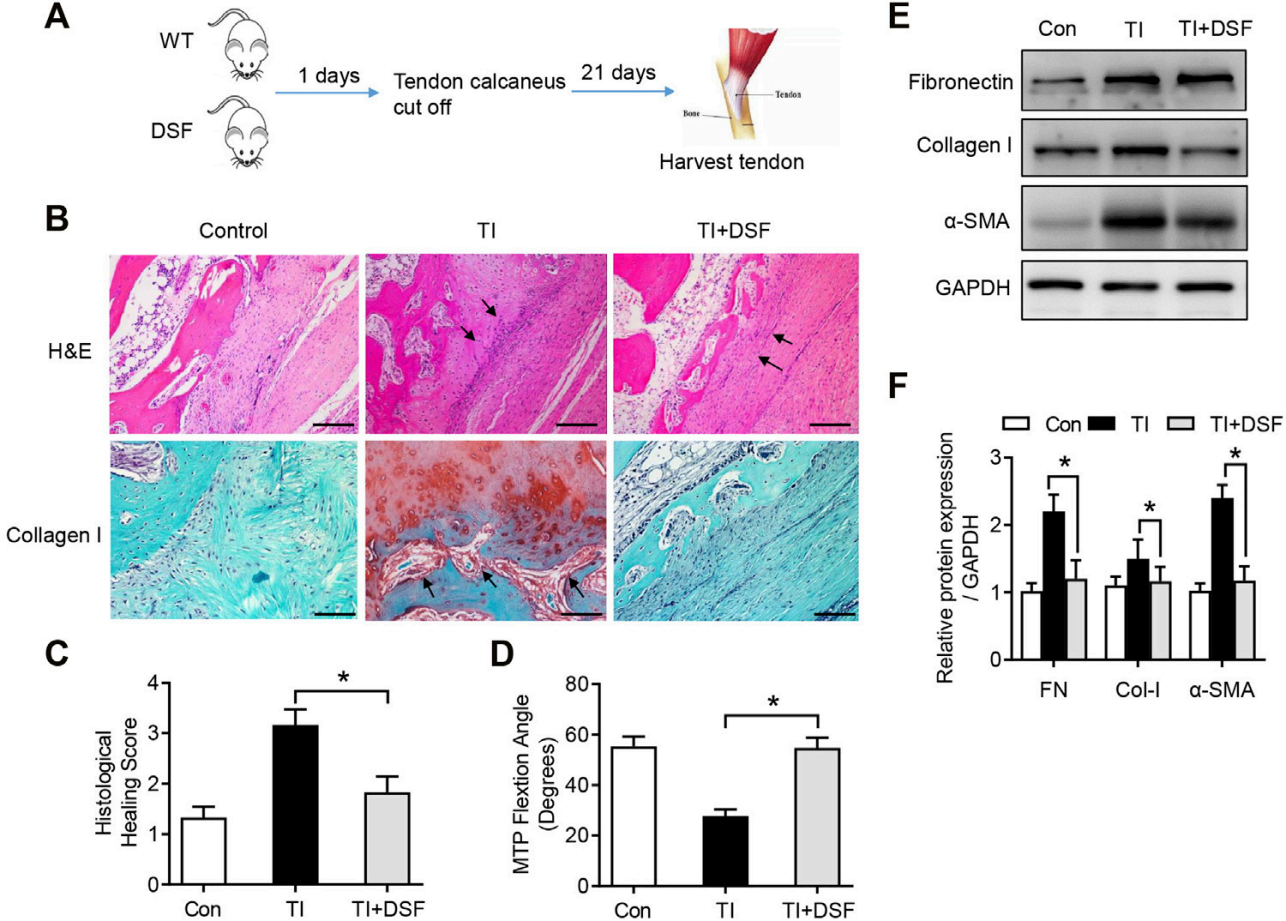
DSF rescued peritendinous fibrosis in TI. **(A)** The diagram of mouse model conduction. **(B)** H&E and immunohistological staining of Col-I in the control, TI, and TI oral with DSF (50 mg/kg) mouse groups. Scale bar = 200 μm. **(C)** Histological adhesion score and histological healing score from the indicated groups (*n* = 6 per group). **(D)** MTP joint flexion ROM of the indicated groups (*n* = 6 per group). **(E)** Western blot analysis of FN, Col-I, and α-SMA in tendon (*n* = 6 per group). Data are presented as the mean ± SEM (*n* = 6) **(C**,**D)**. *p < 0.05.

To assess the possible role of DSF in peritendinous fibrosis following TI, mice were treated with DSF through oral administration after tendon surgery. Mice were randomly assigned to the following three groups: sham operation, TI with acetylcellulose, and TI with DSF (Figure 3A). The histological analysis showed significantly reduced adhesion formation and inflammatory cell infiltration and fibroblasts accumulating in the tendon–bone interface in TI mice after DSF treatment (Figure 3B). The immunohistochemical staining for collagen I was also robust in TI mice and significantly suppressed after DSF treatment (Figure 3B). Consistent with these results, the histological adhesion score was markedly higher in the TI than in the TI-with-DSF treatment group, thus indicating decreased fibrogenesis in the latter (Figure 3C). We also measured the metatarsophalangeal (MTP) joint flexion range of motion (ROM) in mice that underwent flexor tendon surgery. There was a significant decrease of the MTP joint ROM in TI mice, which was substantially reduced after DSF treatment (Figure 3D). As peritendinous fibrosis is manifested by the excessive production of ECM proteins FN, collagen type I (Col-I), and α-SMA in TI (Harvey et al., 2019; Liu et al., 2021), we next assessed the expression of these pro-fibrotic markers in tendons by western blot analysis. The results showed increased levels of FN, Col-I, and α-SMA in TI mice compared with the control. However, the increased pro-fibrotic markers were significantly suppressed in TI mice after DSF treatment (Figure 3E). Overall, these results suggested that DSF suppressed peritendinous fibrosis and can serve as a potential therapeutic agent in TI mice.

### DSF Inhibited Pro-inflammatory Cytokine Production and Macrophage Polarization

**FIGURE 4:**
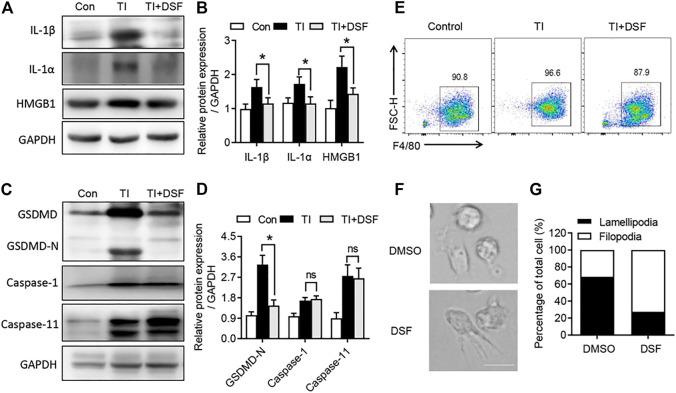
DSF inhibited GSDMD-dependent cell pyroptosis and inflammatory cytokine release. **(A)** Western blot analysis of IL-1β, IL-1α, and HMGB1 in tendon from control, TI, and TI oral with DSF mouse groups. **(B)** Quantitative analysis of IL-1β, IL-1α, and HMGB1 in **A**. **(C)** Western blot analysis of GSDMD, GSDMD-N, caspase-1, and caspase-11 in tendon from the indicated groups. **(D)** Quantitative analysis of GSDMD-N, caspase-1, and caspase-11 in **C**. **(E)** FACS of M-CSF-induced macrophages (F4/80^+^) from control, TI, and TI oral with DSF mouse groups. **(F,G)** BMDMs were pretreated with DMSO or DSF (5 μM) for 2 h. Then the cells were treated with LPS (100 ng/ml), IFN-γ (20 ng/ml), and IL-4 (20 ng/ml) for 6 h. The morphology of macrophages was observed by differential interference-contrast microscopy **(F)** and ratios of lamellipodial to filopodial macrophages **(G)**. Data are presented as the mean ± SEM (*n* = 6) **(B, D)**. *p < 0.05.

DSF has recently been identified as a potent inhibitor of GSDMD-mediated pyroptosis and inflammatory cytokine release (Hu et al., 2020). We next investigated the pro-inflammatory cytokine production and GSDMD pathway in the repaired tendons. The levels of IL-1β, IL-1α, and HMGB1 were significantly increased in TI mice. DSF markedly suppressed the expression of these pro-inflammatory cytokines (Figure 4A). In addition, we observed an increased expression of GSDMD and a detected band of GSDMD-N terminal in TI mice (Figure 4). GSDMD activation has been reported to be mediated by the pyroptotic caspase-1, caspase-4, and caspase-5 in humans and caspase-1 and caspase-11 in mice (Kesavardhana et al., 2020). We then investigated the expression of caspase-1 and caspase-11. Western blot showed that the expression levels of caspase-1 and caspase-11 proteins were increased in TI mice. The cleaved GSDMD-N was significantly suppressed after DSF treatment. However, the expression levels of caspase-1 and caspase-11 proteins were not suppressed by DSF (Figure 4). We also induced BMDMs from the control, TI, and TI + DSF mice. The results indicated that the population of F4/80 in BMDM was suppressed in TI mice after DSF treatment (Figure 4E). To further explore the potential role of DSF on M2 macrophages, we observed altered morphology in *ex vivo* cultured BMDMs after LPS treatment. LPS induced lamellipodia-like structures while DSF-treated macrophages showed filopodia-like structures (Figure 4F). Collectively, these results suggested that DSF significantly suppressed pro-inflammatory cytokine production and M1-type macrophage population in TI mice.

### DSF Suppressed Fibroblast Activation and Proliferation After BMDM Exosome Treatment

**FIGURE 5:**
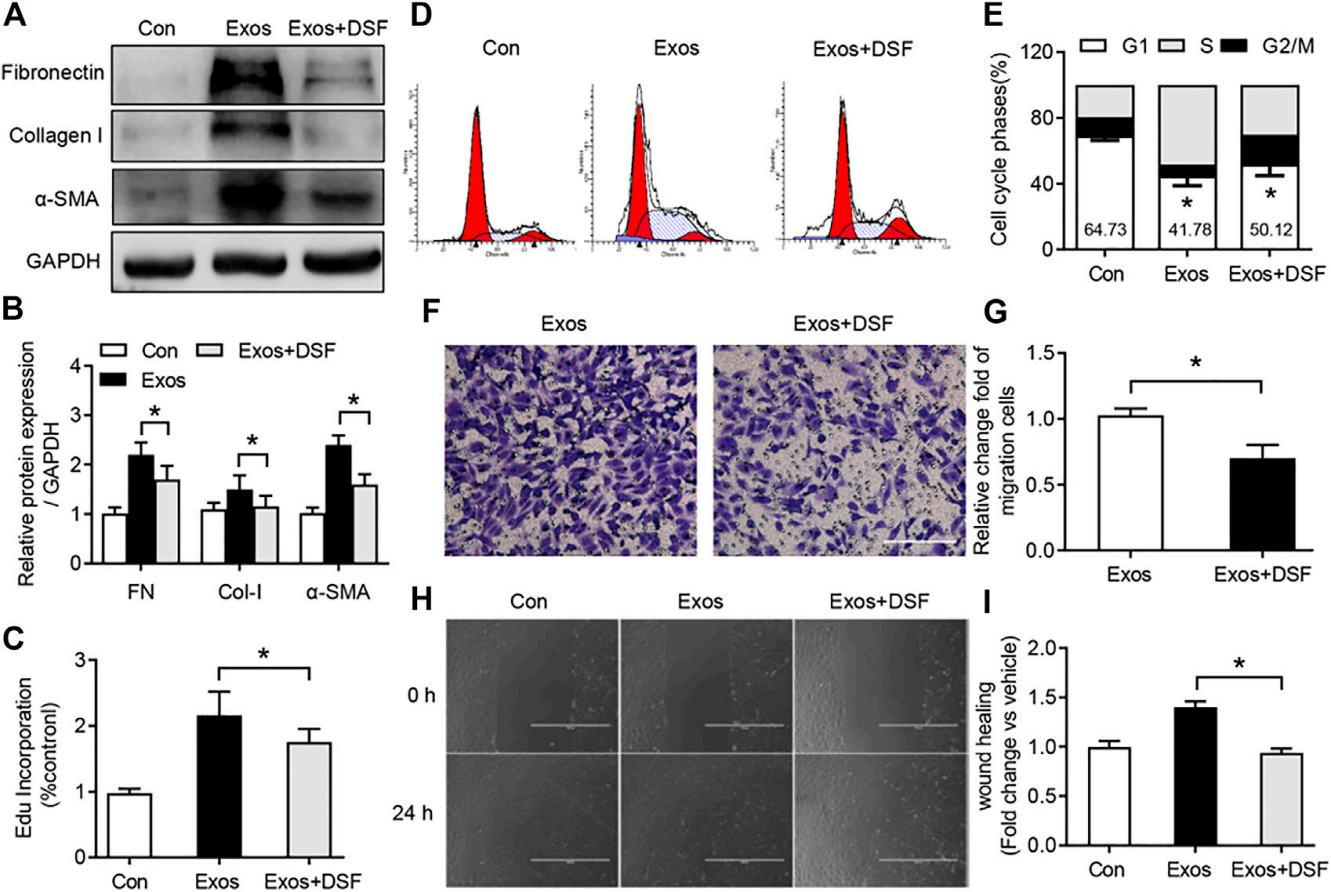
DSF significantly reduced fibroblast activation and proliferation after BMDM exosome treatment. NIH/3T3 fibroblasts were cultured and treated with macrophage-derived exosomes. Cells were pretreated with DSF (5 μM) or DMSO (0.01%) for 2 h. **(A,B)** Western blot **(A)** and quantitative **(B)** analyses of FN, Col-I, and α-SMA in fibroblast from control, exosomes, and exosomes in the presence of DSF. **(C)** Quantitative analysis of EdU incorporation in fibroblast from control, exosomes, and exosomes in the presence of DSF. **(D,E)** FACS **(D)** and quantitative **(E)** analysis of cell cycle in fibroblast from control, exosomes, and exosomes in the presence of DSF. **(F,G)** The picture **(F)** and quantitative analysis **(G)** of the cell migration in fibroblast from control, exosomes, and exosomes in the presence of DSF. Scale bar = 20 μm. **(H,I)** The picture **(H)** and quantitative analysis **(I)** of the cell wound scratch assay in fibroblast from control, exosomes, and exosomes in the presence of DSF. Scale bar = 20 μm. Data are presented as the mean ± SEM (*n* = 3) **(B,C,E,G,I)**. *p < 0.05.

Excessive fibroblast proliferation is an important pathological characteristic of peritendinous fibrosis after TI (Zheng et al., 2017). A number of recent studies have indicated that macrophage-derived exosomes can promote proliferation, migration, and fibrotic activity of fibroblast (Cui et al., 2019). To determine whether DSF was required for fibroblast proliferation and activity after BMDM exosome treatment, NIH/3T3 fibroblasts were cultured and treated with macrophage-derived exosomes. Western blot analysis showed increased FN, Col-I, and α-SMA expression in the cultured fibroblast with BMDM exosome treatment, while DSF significantly suppressed the expression of these pro-fibrotic markers (Figure 5). Subsequent analysis of proliferation revealed a significant upregulation of EdU in the fibroblast after BMDM exosome treatment (Figure 5C), which was attenuated after DSF treatment. Moreover, the increased phase of G2/M plus S after BMDM exosome treatment was significantly suppressed by DSF (Figure 5). We further detected the migration capacity of fibroblast by crystal violet staining (Figure 5) and scratch test (Figure 5). The increased upregulation of migration capacity of fibroblast after BMDM exosome treatment was found to be significantly suppressed by DSF. These *in vitro* data support evidence for a pro-fibrogenic role of BMDM exosomes in fibroblast, and DSF can be an effective therapeutic agent for modulating BMDM exosome-induced fibroblast proliferation and migration.

## Discussion

The communication between macrophages and peritendinous fibroblast plays an essential role in the tendon-healing process. Presently, there is considerable interest in tendon–bone injury that can specifically target macrophage activity, such as macrophage-derived miRNA, exosomes, or chemokines (Gelberman et al., 2017; Cui et al., 2019; Lehner et al., 2019). In this study, we have revealed a critical role of DSF in macrophage polarization and peritendinous fibrosis. In addition, a significant reduction in the levels of various pro-inflammatory cytokines such as IL-1β, TNF-α, and HMGB1 in TI mice was noted after DSF treatment. We also found that DSF significantly suppressed fibroblast activation and proliferation after BMDM exosome treatment. Furthermore, we detected the cleaved GSDMD-N and pro-inflammatory cytokines in the healing tendon of TI mice. These findings reveal the possible involvement of a novel pathway of GSDMD-dependent macrophage cell pyroptosis in remodeling fibrogenesis in tendon–bone injury. GSDMD inhibitor DSF may thus function as potential therapeutic agent in tendon–bone injury.

Infiltration of macrophages to cause tendon–bone damage has recently been increasingly associated with peritendinous adhesion (Cai et al., 2021). In an *in vitro* study, M-CSF-induced BMDMs displayed an increased F4/80 staining in the TI mice than in the control. Through systematically screening a compound library, we found that DSF significantly suppressed pro-inflammatory-type macrophages and facilitated the transformation of M2-type macrophages as indicated by increased expression levels of CD86 and CD206. The results suggested that DSF can be a therapeutic target of immune cells in remodeling tendon–bone injury. It has been established that the initiation of tendon pathology depends upon immune cell infiltration and pro-inflammatory cytokine production, such as IL-6 and TNF-α (Kawamura et al., 2005; Stauber et al., 2021). In addition, TI can elicit an increase in macrophage accumulation from 1 to 28 days in Achilles TI (Marsolais et al., 2001). Furthermore, it has been reported that there was an increased infiltration of macrophages in torn supraspinatus tendon from human tendinopathy (Millar et al., 2010), and the NF-κB pathway was found to be upregulated in fibrous tissue formed around the tendon in both human and rat samples (Chen et al., 2017). Moreover, although macrophages can regulate the TI through releasing several pro-inflammatory cytokines, growth factors, or exosomes, whether there are any small-molecular compounds that can potentially mediate the crosstalk between macrophage and peritendinous fibroblast is not fully understood.

For an *in vivo* study, we have used a mouse model wherein mice underwent transection and repair of the FDL tendon in the right hind paw. This mouse model has a peritendinous fibrosis formation around the injured tendon (Ackerman and Loiselle, 2016; Lin et al., 2020). The results of our study also showed increased F4/80-positive stained cells around α-SMA cells, which clearly indicated the mutual activation of both macrophage and fibroblast. A number of previous studies have identified that macrophages are necessary for peritendinous fibrosis in this mouse model by facilitating macrophage depletion with clodronate liposomes (Cui et al., 2019). This enabled us to investigate whether inflammatory cytokines or damage-associated molecular patterns (DAMPs) that are released from macrophages might play an essential role in TI mice. The data showed that the levels of macrophage-derived IL-1β, IL-1α, or HMGB1 were significantly increased in healing tendon. The significant abundance of IL-1β, IL-1α, or HMGB1 in healing tendon may be attributed to enhanced inflammatory response, which can increase the accumulation of macrophage to the injured site (Gelberman et al., 2017; Li et al., 2021). Our immunofluorescence data also indicated increased macrophage infiltration, accompanied with peritendinous fibroblast activation in tendon bone injury, thereby suggesting that BMDM can serve as important mediators of the peritendinous fibrosis. The regulation of the pro-inflammatory phenotype macrophage transformation may act as an important messenger in mediating intercellular crosstalk between immune cells and resident tenocyte or fibroblast.

Although DSF has been used as an FDA-approved drug for treating alcohol addiction, recent studies have revealed its novel biological effects including antitumor and antimetabolic activities in diverse models (Author Anonymous, 2018; Bernier et al., 2020). In our *in vivo* study, we demonstrated that DSF alleviated peritendinous tissue fibrosis lesions after the TI. It has been suggested that the application of DSF may be limited *in vivo* due to low water solubility, poor stability, and rapid metabolism. In live animals, orally administered DSF can be metabolized into dithiocarbamate (DTC) or DSF/Cu complex (Zhou et al., 2018; Wu et al., 2019), and it has been identified that the DSF/Cu complex can directly bind to nuclear protein localization 4 (NPL4) and promote cell apoptosis (Skrott et al., 2017). So further studies are needed to identify whether DTC or DSF/Cu complex can act as important mediators in TI. Our *in vitro* data showed that DSF exhibited a potent antifibrotic effect by, modulating the expression of various fibrotic markers such as FN, Col-I, and α-SAM in fibroblasts after BMDM exosome treatment. BMDM exosomes have previously been shown to promote fibrosis in tendon–bone injury (Cui et al., 2019). Our studies also demonstrated that DSF can significantly reduce the cell proliferation and migration after BMDM exosome treatment. These results indicated that there might be distinct effects of DSF on both the macrophages and fibroblasts. Since DSF dysregulated the p97-NPL4-ubiquitin fusion degradation protein 1 (UFD1) pathway, the effect of DSF on fibroblast proliferation and migration may be primarily dependent on the erroneous protein accumulation and cell death. Further studies are required to investigate the precise mechanism of action of DSF on fibroblasts.

GSDMD has been recently identified as a pyroptotic executioner, which can participate in the numerous diseases, such as acute kidney injury, alcoholic hepatitis, or neuroinflammation (Khanova et al., 2018; Li et al., 2019; Miao et al., 2019). Our results showed that the cleaved N-terminal of GSDMD was predominantly detected in tendon–bone injury, and the level of IL-1β was significantly suppressed after DSF treatment. In recent years, expression and activation of GSDMD have been reported in macrophages, and GSDMD-dependent pore formation can act as a conduit for IL-1β secretion (Heilig et al., 2018). GSDMD contains the N-terminal GSDMD-N domain and the C-terminal GSDMD-C domain, which are linked together by a long loop (Shi et al., 2017). The cleaved GSDMD-N thus oligomerizes in the membranes, causes lytic cells, and stimulates IL-1β release. GSDMD is widely expressed in the different tissues and cell types, with high levels of expression in the macrophages and gastrointestinal epithelia (Saeki et al., 2000). Since the whole protein in western blot contains tenocytes, fibroblasts, and macrophages, further investigations are needed to identify whether GSDMD can also be expressed in tenocyte cells and whether tenocyte cells can undergo pyroptosis during the TI.

In conclusion, the data generated in the present study highlighted an important role of DSF-mediated macrophage polarization as a potential driver of TI mice. We found that TI can trigger the upregulation of different pro-inflammatory cytokines. Our results also suggest an essential role of DSF for peritendinous fibrosis. Moreover, the FDA-approved drug DSF, which is used to treat alcohol addiction, has been recently shown to inhibit GSDMD pore formation. We found that DSF was able to effectively inhibit pyroptosis and IL-1β release from M1-type macrophages. Given the significant unmet clinical needs of TI patients, the therapeutic potential of the repurposing of this drug as a means of treating TI warrants further studies.

## Data Availability

The raw data supporting the conclusion of this article will be made available by the authors, without undue reservation.
