# Corrigendum: Disulfiram suppressed peritendinous fibrosis through inhibiting macrophage accumulation and its pro-inflammatory properties in tendon bone healing

**DOI:** 10.3389/fbioe.2022.1054283

**Published:** 2022-11-10

**Authors:** Qi Zhou, Wei Wang, Fujun Yang, Hao Wang, Xiaodong Zhao, Yiqin Zhou, Peiliang Fu, Yaozeng Xu

**Affiliations:** ^1^ Department of Orthopedics, the First Affiliated Hospital of Soochow University, Suzhou, China; ^2^ Department of Orthopedics, Shanghai Changzheng Hospital, Naval Medical University, Shanghai, China; ^3^ The Fifth People’s Hospital of Zunyi, Zunyi, China; ^4^ Department of Orthopaedics, Shanghai Public Health Clinical Center, Fudan University, Shanghai, China; ^5^ Department of Orthopaedics, Weifang Traditional Chinese Hospital, Weifang, China

**Keywords:** macrophages, tendon-bone injury, disulfiram, gasdermin D, fibrosis

In the published article, there was an error in Figure 2C as published. The CD206 flow data in the DSF group has been corrected**.** The corrected Figure 2C and its caption appear below.

**FIGURE 2:**
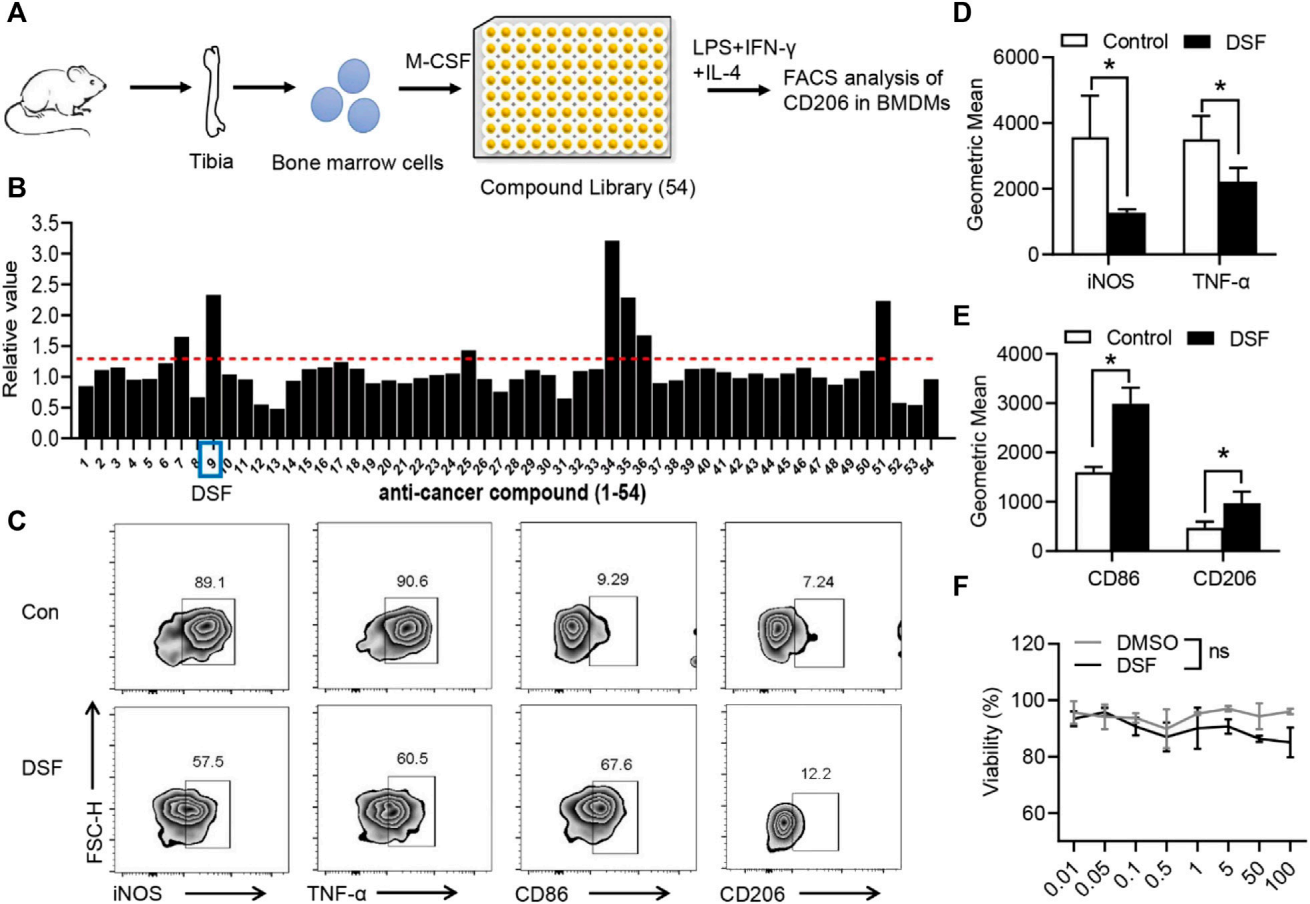
DSF significantly promotes pro-inflammatory macrophages into the M2-type phenotype. **(A)** The diagram of screening assay. Bone marrow cells were isolated from the tibia of wild-type mice. BMDMs were induced by M-CSF (50 ng/ml). Then the cells were treated with LPS (100 ng/ml), IFN-γ (20 ng/ml), and IL-4 (20 ng/ml) for 6 h and underwent FACS analysis of CD206 expression. **(B)** Quantitative analysis of the candidates in screening assay. **(C)** BMDMs were pretreated with DSF (5 μM) or DMSO (0.01%). Then the cells were treated with LPS (100 ng/ml), IFN-γ (20 ng/ml), and IL-4 (20 ng/ml) for 6 h. FACS analysis of iNOS, TNF-α, CD86, and CD206. **(D,E)** Quantitative analysis of geometric mean of iNOS **(D)**, TNF-α **(D)**, CD86 **(E)**, and CD206 **(E)**. **(F)** CCK-8 analysis of BMDMs after treatment with DSF at different dosages. Data are presented as the mean ± SEM (*n* = 3) **(D-F)**. *p < 0.05.

Additionally, in the published article, there was an textual error. A correction has been made to **Results**, “DSF decreased macrophage pro-inflammatory phenotype and can promote the transition of macrophage from M1 to M2 phenotype”, **paragraph 1**.

This sentence previously stated:

“while the expression levels of CD206 and Arg1”

The corrected sentence appears below:

“while the expression levels of CD86 and CD206”

The authors apologize for these errors and state that they do not change the scientific conclusions of the article in any way. The original article has been updated.

